# 2-(2-Hydroxyphenyl)-4,5-dimethyl-1*H*-imidazol-3-ium acetate monohydrate

**DOI:** 10.1107/S1600536808028626

**Published:** 2008-09-17

**Authors:** Hui-Liang Wen, Min He, Chong-Bo Liu

**Affiliations:** a Department of Chemistry, Nanchang University, Nanchang 330031, People’s Republic of China; b State Key Laboratory of Food Science and Technology, Nanchang University, Nanchang 330047, People’s Republic of China; c College of Environmental and Chemical Engineering, Nanchang University of Aeronautics, Nanchang 330063, People’s Republic of China

## Abstract

In the title compound, C_11_H_13_N_2_O^+^·C_2_H_3_O_2_^−^·H_2_O, the dihedral angle between the benzene ring and the imidazole ring is 7.83 (6)°. In the crystal structure, N—H⋯O and O—H⋯O hydrogen bonds form a two-dimensional network. All the methyl H atoms are disorderd over two sites with equal occupancies.

## Related literature

For related literature, see: Maeda *et al.* (1984 ▶); Puratchikody & Doble (2007 ▶); Quattara *et al.* (1987 ▶); Ucucu *et al.* (2001 ▶); Scott *et al.* (2004 ▶); Seko *et al.* (1991 ▶).
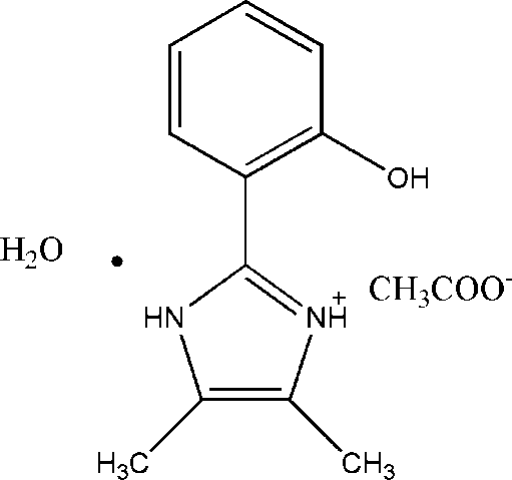


## Experimental

### 

#### Crystal data

C_11_H_13_N_2_O^+^·C_2_H_3_O_2_^−^·H_2_O
*M* *_r_* = 266.29
Monoclinic, 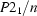

*a* = 8.1655 (12) Å
*b* = 9.6542 (14) Å
*c* = 17.141 (3) Å
β = 96.374 (2)°
*V* = 1342.9 (3) Å^3^
*Z* = 4
Mo *K*α radiation
μ = 0.10 mm^−1^
*T* = 295 (2) K
0.46 × 0.38 × 0.24 mm

#### Data collection

Bruker SMART CCD diffractometer
Absorption correction: multi-scan (*SADABS*; Sheldrick, 1996 ▶) *T*_min_ = 0.956, *T*_max_ = 0.977
8442 measured reflections
2488 independent reflections
1751 reflections with *I* > 2σ(*I*)
*R*_int_ = 0.032

#### Refinement

*R*[*F* ^2^ > 2σ(*F* ^2^)] = 0.041
*wR*(*F* ^2^) = 0.109
*S* = 1.05
2488 reflections
174 parameters
H-atom parameters constrained
Δρ_max_ = 0.20 e Å^−3^
Δρ_min_ = −0.15 e Å^−3^

### 

Data collection: *SMART* (Bruker, 1998 ▶); cell refinement: *SAINT* (Bruker, 1998 ▶); data reduction: *SAINT*; program(s) used to solve structure: *SHELXS97* (Sheldrick, 2008 ▶); program(s) used to refine structure: *SHELXL97* (Sheldrick, 2008 ▶); molecular graphics: *SHELXTL* (Sheldrick, 2008 ▶); software used to prepare material for publication: *SHELXTL*.

## Supplementary Material

Crystal structure: contains datablocks global, I. DOI: 10.1107/S1600536808028626/lh2684sup1.cif

Structure factors: contains datablocks I. DOI: 10.1107/S1600536808028626/lh2684Isup2.hkl

Additional supplementary materials:  crystallographic information; 3D view; checkCIF report

## Figures and Tables

**Table 1:** Hydrogen-bond geometry (Å, °)

*D*—H⋯*A*	*D*—H	H⋯*A*	*D*⋯*A*	*D*—H⋯*A*
N2—H2*D*⋯O4	0.86	1.93	2.7747 (19)	169
N1—H1*D*⋯O1	0.86	2.17	2.6956 (19)	119
N1—H1*D*⋯O3	0.86	2.10	2.834 (2)	142
O4—H2*W*⋯O3^i^	0.84	1.89	2.710 (2)	164
O4—H1*W*⋯O2^ii^	0.84	2.07	2.808 (2)	146
O1—H1⋯O2^iii^	0.82	1.76	2.5624 (18)	167

Symmetry codes: (i) 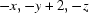; (ii) 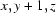; (iii) 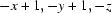.

**Fig. 1.**
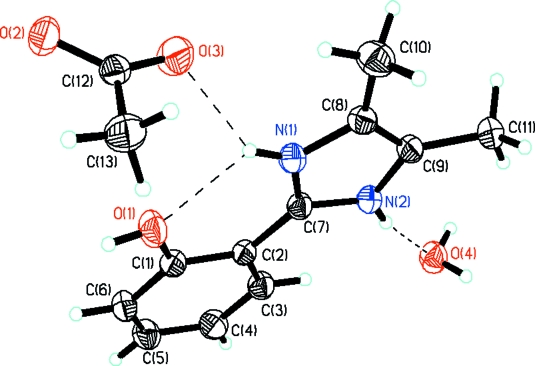
: The molecular structure of the title compound with the atom-labelling scheme. Displacement ellipsoids are drawn at the 30% probability level and H atoms are represented as spheres of arbitrary radii. Hydrogen bonds are shown as a dashed lines. Only one disorder site for each methyl H atom is showm.

**Fig. 2.**
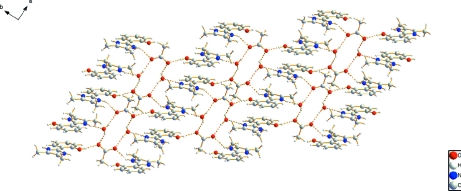
: Part of the crystal structure with hydrogen bonds shown as dashed lines.

## supplementary crystallographic information

### Comment

Imidazole derivatives can have a wide range of biological activities such as analgesic (Ucucu *et al.*, 2001), antiinflammmatory (Maeda *et al.*, 1984), antiparasitic (Quattara *et al.*, 1987), antiepileptic and platelet aggregation inhibitors (Seko *et al.*, 1991). The neutral imidizole component of the title compound could potentially exhibit biological activities (Puratchikody & Doble, 2007). In this paper, we report the crystal structure of the title compound (I).

In the title compound (Fig. 1), the benzene ring and the imidazole ring are approximately co-planar with a dihedral angle of 7.83 (6)° between them. The components of the salt are linked via N-H···O hydrogen bonds. In the crystal structure, intermolecular O—H···O and N—H···O hydrogen bonds link the components of the title compound into a two-dimensional network.

### Experimental

The title compound was prepared according to a literature method (Scott *et al.*, 2004). 1.72 g (20 mmol) butane-2,3-dione, 2.44 g (20 mmol), 4-hydroxybenzaldehyde and 5 g (>50 mmol) NH_4_Ac were placed in a sealed container with 100 ml CH_3_Cl:HAc (4:1) as the solvent and heated in a micro-wave at 350 W for 24 min. After the reaction, the solvent was evaporated. The pure product as a dark red crystalline solid was obtained by re-crystallization from hot EtOH/H_2_O in a yield of 81.6% and suitable for X-ray diffraction analysis.

### Refinement

The water H atoms were located in a difference Fourier map and refined with idealized calculated O—H distances of 0.84Å and *U*_iso_(H) = 1.5 *U*_eq_(O). All other H atoms were placed at geometrically idealized positions with C—H (methyl) = 0.96 Å and C—H = 0.93Å for phenyl, N—H = 0.86 Å, and *U*_iso_(H) = 1.2 *U*_eq_(C,*N*). All methyl H atoms were refined as disorded over two sites with 0.5 occupancy.

### Crystal data

**Table:**

C_11_H_13_N_2_O^+^·C_2_H_3_O_2_^−^·H_2_O	*F*(000) = 568
*M**_r_* = 266.29	*D*_x_ = 1.317 Mg m^−^^3^
Monoclinic, *P*2_1_/*n*	Mo *K*α radiation, λ = 0.71073 Å
Hall symbol: -P 2yn	Cell parameters from 1719 reflections
*a* = 8.1655 (12) Å	θ = 2.4–21.9°
*b* = 9.6542 (14) Å	µ = 0.10 mm^−^^1^
*c* = 17.141 (3) Å	*T* = 295 K
β = 96.374 (2)°	Block, yellow
*V* = 1342.9 (3) Å^3^	0.46 × 0.38 × 0.24 mm
*Z* = 4	

### Data collection

**Table:**

Bruker SMART CCD diffractometer	2488 independent reflections
Radiation source: fine-focus sealed tube	1751 reflections with *I* > 2σ(*I*)
graphite	*R*_int_ = 0.032
φ and ω scans	θ_max_ = 25.5°, θ_min_ = 2.4°
Absorption correction: multi-scan (SADABS; Sheldrick, 1996)	*h* = −9→9
*T*_min_ = 0.956, *T*_max_ = 0.977	*k* = −11→11
8442 measured reflections	*l* = −20→20

### Refinement

**Table:**

Refinement on *F*^2^	Secondary atom site location: difference Fourier map
Least-squares matrix: full	Hydrogen site location: inferred from neighbouring sites
*R*[*F*^2^ > 2σ(*F*^2^)] = 0.041	H-atom parameters constrained
*wR*(*F*^2^) = 0.109	*w* = 1/[σ^2^(*F*_o_^2^) + (0.0429*P*)^2^ + 0.2995*P*] where *P* = (*F*_o_^2^ + 2*F*_c_^2^)/3
*S* = 1.05	(Δ/σ)_max_ < 0.001
2488 reflections	Δρ_max_ = 0.20 e Å^−^^3^
174 parameters	Δρ_min_ = −0.15 e Å^−^^3^
0 restraints	Extinction correction: SHELXL, Fc^*^=kFc[1+0.001xFc^2^λ^3^/sin(2θ)]^-1/4^
Primary atom site location: structure-invariant direct methods	Extinction coefficient: 0.0109 (16)

### Special details

**Table:**

Geometry. All e.s.d.'s (except the e.s.d. in the dihedral angle between two l.s. planes) are estimated using the full covariance matrix. The cell e.s.d.'s are taken into account individually in the estimation of e.s.d.'s in distances, angles and torsion angles; correlations between e.s.d.'s in cell parameters are only used when they are defined by crystal symmetry. An approximate (isotropic) treatment of cell e.s.d.'s is used for estimating e.s.d.'s involving l.s. planes.
Refinement. Refinement of *F*^2^ against ALL reflections. The weighted *R*-factor *wR* and goodness of fit *S* are based on *F*^2^, conventional *R*-factors *R* are based on *F*, with *F* set to zero for negative *F*^2^. The threshold expression of *F*^2^ > σ(*F*^2^) is used only for calculating *R*-factors(gt) *etc*. and is not relevant to the choice of reflections for refinement. *R*-factors based on *F*^2^ are statistically about twice as large as those based on *F*, and *R*- factors based on ALL data will be even larger.

### Fractional atomic coordinates and isotropic or equivalent isotropic displacement parameters (Å^2^)

**Table:**

	*x*	*y*	*z*	*U*_iso_*/*U*_eq_	Occ. (<1)
O1	0.43862 (18)	0.76646 (13)	−0.03904 (8)	0.0587 (4)	
H1	0.5030	0.7086	−0.0529	0.088*	
O3	0.24781 (18)	0.61838 (14)	0.08451 (11)	0.0777 (5)	
O2	0.33771 (17)	0.41072 (13)	0.06150 (9)	0.0600 (4)	
O4	0.08440 (17)	1.35614 (13)	−0.05919 (8)	0.0593 (4)	
H1W	0.1378	1.4036	−0.0243	0.089*	
H2W	−0.0162	1.3770	−0.0610	0.089*	
N1	0.25223 (18)	0.90693 (15)	0.05447 (9)	0.0435 (4)	
H1D	0.2938	0.8251	0.0545	0.052*	
N2	0.17344 (18)	1.11013 (14)	0.01783 (9)	0.0411 (4)	
H2D	0.1545	1.1834	−0.0103	0.049*	
C1	0.4058 (2)	0.86127 (18)	−0.09630 (11)	0.0425 (4)	
C2	0.3119 (2)	0.97773 (17)	−0.08056 (10)	0.0385 (4)	
C3	0.2787 (2)	1.07700 (19)	−0.13939 (11)	0.0467 (5)	
H3	0.2185	1.1555	−0.1293	0.056*	
C4	0.3333 (3)	1.0612 (2)	−0.21208 (12)	0.0546 (5)	
H4	0.3099	1.1282	−0.2507	0.066*	
C5	0.4233 (3)	0.9446 (2)	−0.22712 (12)	0.0562 (6)	
H5	0.4589	0.9326	−0.2764	0.067*	
C6	0.4602 (2)	0.8466 (2)	−0.17006 (12)	0.0521 (5)	
H6	0.5223	0.7695	−0.1807	0.063*	
C7	0.2492 (2)	0.99653 (17)	−0.00505 (10)	0.0388 (4)	
C8	0.1793 (2)	0.96438 (19)	0.11599 (11)	0.0444 (5)	
C9	0.1301 (2)	1.09338 (18)	0.09268 (11)	0.0429 (5)	
C10	0.1708 (3)	0.8898 (2)	0.19128 (12)	0.0630 (6)	
H10A	0.2175	0.7991	0.1880	0.095*	0.50
H10B	0.0578	0.8818	0.2013	0.095*	0.50
H10C	0.2314	0.9405	0.2332	0.095*	0.50
H10D	0.1203	0.9485	0.2270	0.095*	0.50
H10E	0.2800	0.8658	0.2137	0.095*	0.50
H10F	0.1064	0.8071	0.1818	0.095*	0.50
C11	0.0489 (3)	1.2060 (2)	0.13390 (12)	0.0574 (6)	
H11A	0.0308	1.2846	0.0997	0.086*	0.50
H11B	0.1186	1.2325	0.1803	0.086*	0.50
H11C	−0.0547	1.1735	0.1482	0.086*	0.50
H11D	0.0323	1.1758	0.1858	0.086*	0.50
H11E	−0.0555	1.2279	0.1052	0.086*	0.50
H11F	0.1178	1.2869	0.1373	0.086*	0.50
C12	0.3592 (2)	0.53088 (18)	0.08843 (11)	0.0448 (5)	
C13	0.5261 (3)	0.5695 (2)	0.12663 (14)	0.0641 (6)	
H13F	0.6052	0.5599	0.0895	0.096*	0.50
H13E	0.5559	0.5097	0.1707	0.096*	0.50
H13D	0.5248	0.6638	0.1443	0.096*	0.50
H13C	0.5187	0.5957	0.1802	0.096*	0.50
H13B	0.5680	0.6459	0.0990	0.096*	0.50
H13A	0.5991	0.4918	0.1253	0.096*	0.50

### Atomic displacement parameters (Å^2^)

**Table:**

	*U*^11^	*U*^22^	*U*^33^	*U*^12^	*U*^13^	*U*^23^
O1	0.0687 (10)	0.0470 (8)	0.0630 (9)	0.0195 (7)	0.0194 (8)	0.0032 (7)
O3	0.0517 (9)	0.0506 (9)	0.1328 (15)	0.0125 (7)	0.0196 (10)	0.0043 (9)
O2	0.0515 (9)	0.0434 (8)	0.0842 (11)	0.0017 (6)	0.0039 (7)	−0.0110 (7)
O4	0.0526 (8)	0.0550 (8)	0.0698 (10)	0.0083 (7)	0.0045 (7)	−0.0070 (7)
N1	0.0426 (9)	0.0386 (8)	0.0498 (10)	0.0050 (7)	0.0073 (7)	0.0003 (7)
N2	0.0427 (9)	0.0363 (8)	0.0449 (9)	0.0021 (7)	0.0073 (7)	−0.0021 (7)
C1	0.0399 (10)	0.0394 (10)	0.0482 (11)	−0.0008 (8)	0.0054 (9)	−0.0036 (8)
C2	0.0358 (10)	0.0358 (9)	0.0443 (11)	−0.0021 (7)	0.0061 (8)	−0.0057 (8)
C3	0.0444 (11)	0.0444 (11)	0.0513 (12)	0.0015 (8)	0.0054 (9)	−0.0031 (9)
C4	0.0615 (13)	0.0566 (12)	0.0460 (12)	−0.0016 (10)	0.0074 (10)	0.0015 (9)
C5	0.0599 (13)	0.0641 (13)	0.0463 (12)	−0.0030 (11)	0.0128 (10)	−0.0110 (10)
C6	0.0529 (12)	0.0492 (11)	0.0558 (13)	0.0038 (9)	0.0130 (10)	−0.0116 (10)
C7	0.0350 (10)	0.0362 (9)	0.0451 (11)	0.0007 (7)	0.0044 (8)	−0.0042 (8)
C8	0.0411 (11)	0.0474 (11)	0.0455 (11)	−0.0026 (8)	0.0078 (9)	−0.0026 (9)
C9	0.0387 (10)	0.0456 (11)	0.0452 (11)	−0.0028 (8)	0.0080 (8)	−0.0074 (8)
C10	0.0687 (15)	0.0692 (14)	0.0525 (13)	0.0011 (11)	0.0132 (11)	0.0092 (10)
C11	0.0586 (13)	0.0541 (12)	0.0624 (13)	−0.0034 (10)	0.0197 (11)	−0.0159 (10)
C12	0.0430 (11)	0.0398 (11)	0.0532 (12)	0.0012 (9)	0.0117 (9)	0.0049 (9)
C13	0.0551 (14)	0.0678 (14)	0.0682 (15)	−0.0103 (11)	0.0010 (11)	−0.0017 (12)

### Geometric parameters (Å, °)

**Table:**

O1—C1	1.347 (2)	C8—C9	1.355 (2)
O1—H1	0.8200	C8—C10	1.486 (3)
O3—C12	1.238 (2)	C9—C11	1.492 (2)
O2—C12	1.254 (2)	C10—H10A	0.9600
O4—H1W	0.8364	C10—H10B	0.9600
O4—H2W	0.8428	C10—H10C	0.9600
N1—C7	1.336 (2)	C10—H10D	0.9600
N1—C8	1.383 (2)	C10—H10E	0.9600
N1—H1D	0.8600	C10—H10F	0.9600
N2—C7	1.339 (2)	C11—H11A	0.9600
N2—C9	1.378 (2)	C11—H11B	0.9600
N2—H2D	0.8600	C11—H11C	0.9600
C1—C6	1.393 (3)	C11—H11D	0.9600
C1—C2	1.404 (2)	C11—H11E	0.9600
C2—C3	1.396 (2)	C11—H11F	0.9600
C2—C7	1.455 (2)	C12—C13	1.493 (3)
C3—C4	1.377 (3)	C13—H13F	0.9600
C3—H3	0.9300	C13—H13E	0.9600
C4—C5	1.384 (3)	C13—H13D	0.9600
C4—H4	0.9300	C13—H13C	0.9600
C5—C6	1.370 (3)	C13—H13B	0.9600
C5—H5	0.9300	C13—H13A	0.9600
C6—H6	0.9300		
			
C1—O1—H1	109.5	H10A—C10—H10F	56.3
H1W—O4—H2W	108.9	H10B—C10—H10F	56.3
C7—N1—C8	110.46 (14)	H10C—C10—H10F	141.1
C7—N1—H1D	124.7	H10D—C10—H10F	109.5
C8—N1—H1D	124.8	H10E—C10—H10F	109.5
C7—N2—C9	110.57 (14)	C9—C11—H11A	109.5
C7—N2—H2D	124.7	C9—C11—H11B	109.5
C9—N2—H2D	124.7	H11A—C11—H11B	109.5
O1—C1—C6	122.33 (16)	C9—C11—H11C	109.5
O1—C1—C2	118.22 (16)	H11A—C11—H11C	109.5
C6—C1—C2	119.44 (17)	H11B—C11—H11C	109.5
C3—C2—C1	118.52 (16)	C9—C11—H11D	109.5
C3—C2—C7	119.85 (15)	H11A—C11—H11D	141.1
C1—C2—C7	121.63 (16)	H11B—C11—H11D	56.3
C4—C3—C2	121.41 (18)	H11C—C11—H11D	56.3
C4—C3—H3	119.3	C9—C11—H11E	109.5
C2—C3—H3	119.3	H11A—C11—H11E	56.3
C3—C4—C5	119.36 (19)	H11B—C11—H11E	141.1
C3—C4—H4	120.3	H11C—C11—H11E	56.3
C5—C4—H4	120.3	H11D—C11—H11E	109.5
C6—C5—C4	120.52 (18)	C9—C11—H11F	109.5
C6—C5—H5	119.7	H11A—C11—H11F	56.3
C4—C5—H5	119.7	H11B—C11—H11F	56.3
C5—C6—C1	120.73 (18)	H11C—C11—H11F	141.1
C5—C6—H6	119.6	H11D—C11—H11F	109.5
C1—C6—H6	119.6	H11E—C11—H11F	109.5
N1—C7—N2	106.06 (15)	O3—C12—O2	122.65 (19)
N1—C7—C2	128.32 (15)	O3—C12—C13	118.89 (18)
N2—C7—C2	125.61 (16)	O2—C12—C13	118.46 (17)
C9—C8—N1	106.41 (15)	C12—C13—H13F	109.5
C9—C8—C10	131.30 (17)	C12—C13—H13E	109.5
N1—C8—C10	122.25 (16)	H13F—C13—H13E	109.5
C8—C9—N2	106.49 (15)	C12—C13—H13D	109.5
C8—C9—C11	131.55 (17)	H13F—C13—H13D	109.5
N2—C9—C11	121.95 (17)	H13E—C13—H13D	109.5
C8—C10—H10A	109.5	C12—C13—H13C	109.5
C8—C10—H10B	109.5	H13F—C13—H13C	141.1
H10A—C10—H10B	109.5	H13E—C13—H13C	56.3
C8—C10—H10C	109.5	H13D—C13—H13C	56.3
H10A—C10—H10C	109.5	C12—C13—H13B	109.5
H10B—C10—H10C	109.5	H13F—C13—H13B	56.3
C8—C10—H10D	109.5	H13E—C13—H13B	141.1
H10A—C10—H10D	141.1	H13D—C13—H13B	56.3
H10B—C10—H10D	56.3	H13C—C13—H13B	109.5
H10C—C10—H10D	56.3	C12—C13—H13A	109.5
C8—C10—H10E	109.5	H13F—C13—H13A	56.3
H10A—C10—H10E	56.3	H13E—C13—H13A	56.3
H10B—C10—H10E	141.1	H13D—C13—H13A	141.1
H10C—C10—H10E	56.3	H13C—C13—H13A	109.5
H10D—C10—H10E	109.5	H13B—C13—H13A	109.5
C8—C10—H10F	109.5		
			
O1—C1—C2—C3	179.44 (16)	C9—N2—C7—C2	−179.93 (16)
C6—C1—C2—C3	−1.2 (3)	C3—C2—C7—N1	171.82 (17)
O1—C1—C2—C7	−0.6 (3)	C1—C2—C7—N1	−8.1 (3)
C6—C1—C2—C7	178.69 (16)	C3—C2—C7—N2	−7.3 (3)
C1—C2—C3—C4	1.3 (3)	C1—C2—C7—N2	172.81 (16)
C7—C2—C3—C4	−178.65 (17)	C7—N1—C8—C9	0.1 (2)
C2—C3—C4—C5	−0.2 (3)	C7—N1—C8—C10	−177.72 (17)
C3—C4—C5—C6	−1.0 (3)	N1—C8—C9—N2	0.34 (19)
C4—C5—C6—C1	1.1 (3)	C10—C8—C9—N2	177.9 (2)
O1—C1—C6—C5	179.40 (18)	N1—C8—C9—C11	−178.04 (19)
C2—C1—C6—C5	0.1 (3)	C10—C8—C9—C11	−0.4 (4)
C8—N1—C7—N2	−0.59 (19)	C7—N2—C9—C8	−0.7 (2)
C8—N1—C7—C2	−179.82 (17)	C7—N2—C9—C11	177.84 (17)
C9—N2—C7—N1	0.82 (19)		

### Hydrogen-bond geometry (Å, °)

**Table:**

*D*—H···*A*	*D*—H	H···*A*	*D*···*A*	*D*—H···*A*
N2—H2D···O4	0.86	1.93	2.7747 (19)	169.
N1—H1D···O1	0.86	2.17	2.6956 (19)	119.
N1—H1D···O3	0.86	2.10	2.834 (2)	142.
O4—H2W···O3^i^	0.84	1.89	2.710 (2)	164.
O4—H1W···O2^ii^	0.84	2.07	2.808 (2)	146.
O1—H1···O2^iii^	0.82	1.76	2.5624 (18)	167.

Symmetry codes: (i) −*x*, −*y*+2, −*z*; (ii) *x*, *y*+1, *z*; (iii) −*x*+1, −*y*+1, −*z*.
